# Assessment of cultivation parameters influencing pectinase production by *Aspergillus niger* LFP-1 in submerged fermentation

**DOI:** 10.1186/s43141-023-00510-z

**Published:** 2023-04-24

**Authors:** Mohd Taufiq Mat Jalil, Nurul Aili Zakaria, Nor Hawani Salikin, Darah Ibrahim

**Affiliations:** 1 School of Biology, Faculty of Applied Sciences, Universiti Teknologi MARA, 40450 Shah Alam, Selangor Malaysia; 2 Bioprocess Technology Division, School of Industrial Technology, Universiti Sains Malaysia, 11800 Minden, Penang Malaysia; 3 Industrial Biotechnology Research Laboratory, School of Biological Sciences, Universiti Sains Malaysia, 11800 Minden, Penang Malaysia

**Keywords:** Pectinase, *Aspergillus niger*, Physiochemical parameters, Submerged fermentation

## Abstract

**Background:**

Pectinase is helpful in food and beverage industries, particularly in the preparation of fruit juice, the extraction of vegetable oil, and the fermentation of coffee. The current work aimed to screen *Aspergillus niger* LFP-1, a recently identified fungal strain, for its ability to produce pectinase and to ascertain the contribution of various physicochemical factors to pectinase production.

**Results:**

The primary and secondary pectinase activity screenings by *Aspergillus niger* LFP-1 were performed using pectin screening agar and shake flask system, respectively. The finding revealed that the locally isolated strain is able to secrete favourable pectinase production. Before improvement, the pectinase production was 0.88 ± 0.09 U/mL. However, the improved conditions such as 6 days of the cultivation period, agitation speed of 150 rpm, inoculum size of 1 × 10^6^ spores/mL, 2.5% (w/v) citrus pectin, and 0.4% (w/v) ammonium nitrate could significantly increase pectinase production up to 7.41 ± 0.24 U/mL, representing an 88% increase. In this study, supplementing 2.5% (w/v) citrus pectin to the culture medium as a carbon source increased enzyme production by up to 3.07 ± 0.17 U/mL. Meanwhile, 0.4% (w/v) ammonium nitrate was used as a nitrogen source yielding the highest enzyme activity with a value of 6.86 ± 0.07 U/mL.

**Conclusion:**

Thus, the locally isolated fungal strain, *A. niger* LFP-1 has outstanding pectinase-producing capability and can be utilized for the commercial production of pectinase. The improved cultural conditions significantly increase pectinase production and shorten the incubation period from 8 days (before improvement) to 6 days (after improvement).

## Background

Pectinases are heterogeneous groups of enzymes that can catalyze the breakdown of pectin. The most studied pectinases are polygalacturonase, pectate lyase, pectin lyase, and pectinesterase [1]. Jayani [2] reported that the group of enzymes is classified based on their mode of action on substrates, and the major classified pectinase is polygalacturonase (PG), pectin lyase (PL), and pectinesterase (PE). Pectinase is useful in industrial applications and is classified as acidic or alkaline depending on the pH required for optimum enzyme activity. Acidic pectinase is most commonly found in the winemaking and fruit juice industries. Alkaline pectinase, on the other hand, is used in the pre-treatment of wastewater from the fruit juice industry, oil extraction, coffee, and tea fermentation, degumming, and retting fiber crops, and virus purification [3].

Pectinase can be produced by a large number of microorganisms including bacteria [3], yeast [4], filamentous fungi [5], and endophytic fungi [6]. However, filamentous fungi such as *Aspergillus niger* were extensively studied among the microbial pectinase since pectinase derived from *A. niger* has been recognized as Generally Recognized as Safe (GRAS) in Opinion Letters by Food and Drug Administration (FDA). Fungal pectinases can be produced through submerged fermentation and solid-state fermentation. Submerged fermentation has been extensively employed due to its short period, low cost, and high yield. Besides, the culture medium and physical conditions can be improved to enhance enzyme production. Many previous studies reported the improvement of physicochemical parameters to increase pectinase activity by fungal strains. For instance, Nsude [7] reported that improved conditions including fermentation period, incubation temperature, initial pH, and the addition of carbon and nitrogen sources have significantly increased pectinase production by *A. niger* in submerged fermentation.

In the present study, we report the production of pectinase by a locally indigenous fungus, *A. niger* LFP-1, which was successfully isolated from rotten oranges. This strain can secrete acidic pectinase and thus, physicochemical parameters were improved to enhance pectinase production. It was noteworthy that the improved culture conditions increased enzyme production and shortened the incubation period to obtain optimal enzyme activity.

## Methods

### Microorganisms, culture maintenance, and inoculum preparation

*Aspergillus niger* LFP-1 which was isolated from rotten oranges was provided by the Industrial Biotechnology Research Laboratory, School of Biological Sciences, Universiti Sains Malaysia, Penang, Malaysia. The fungal culture was maintained on potato dextrose agar slant supplemented with 1.0% (w/v) citrus pectin at 30 °C for 3 days aerobically until sporulated before storing them at 4 °C until further use. The subculturing was done monthly to ensure its survivability and purity.

### Preparation of spore suspension

The spore’s inoculum was prepared by adding 5.0 mL of sterile distilled water containing Tween 80 (0.1%, v/v) to a sporulated culture. The spores were dislodged by shaking vigorously and using a sterile inoculation loop. The spore suspension was adjusted using a hemocytometer chamber (Neubauer Germany) to obtain 1 × 10^7^ spores/mL.

### Cultivation medium

A spore suspension (1 × 10^7^ spores/mL) was inoculated into 250 ml Erlenmeyer flasks containing 100 mL of pectin broth medium [consisted of (NH_4_)_2_SO_4_, 0.2% (w/v); MnSO_4_.7H_2_O, 0.007% (w/v); and citrus pectin, 1.0% (w/v); pH4.5] and incubated at 30 ºC for 10 days with an agitation speed of 150 rpm. The cultures were withdrawn at every 48 h intervals and were assayed for pectinase activity and fungal growth determination.

### Screening of pectinase production

For primary screening, a small fragment of *Aspergillus niger* LFP-1 was point inoculated onto the pectinase agar medium consisting of pectin citrate 10.0 (g/L), urea 0.05 (g/L), ammonium sulfate 0.15 (g/L), and agar 22.0 (g/L) (pH 4.5) and incubated at 30 °C for 3–4 days. After the incubation, a 1.0% (w/v) cetrimide (cetyl trimethyl ammonium bromide) solution was flooded onto the agar to detect the presence of a clear zone around the colony of the isolates [8]. After 30 min, the clear zone was observed, and the diameter of the clear zone was measured. For secondary screening, 1.0 mL of the fungal spore suspension was inoculated into 250 mL Erlenmeyer flasks containing 100 mL pectin broth medium and incubated at 30 °C for 5 days under 150 rpm agitation speed. After incubation, the sample was taken out and filtered using Whatman No. 1 filter paper. The filtrate was used to determine pectinase activity and fungal biomass. The experiments were carried out in triplicate.

### Enzyme recovery and fungal biomass determination

The fermentative broth was filtered through filter paper (Whatman No. 1) to separate the fungal mycelium. The cell-free culture filtrate containing the crude enzyme was then assayed for pectinase activity. The filter paper containing fungal biomass was dried at 80 °C until a constant weight was achieved, and the fungal growth (cell dry weight) was obtained by deducting the weight of the filter paper and the growth was then expressed as g/L. All the experiments were performed in triplicate.

### Pectinase activity

Pectinase assay was carried out by measuring reducing sugars released from pectin hydrolyzation as described in the dinitrosalicylic acid (DNS) method [9]. A 0.5 mL crude enzyme was added to a solution containing 0.5 mL of pectin (1.0%, w/v) in 0.1 M citrate buffer, pH 4.5. After 30 min of incubation period at 45 °C, reducing sugars were determined using galacturonic acid as a reference. The pectinase activity was expressed in terms of Unit (U). One unit of pectinase activity was defined as the amount of pectinase that catalyzes the release of 1 µmol of galacturonic acid per mL of culture filtrate per minute under assay conditions.

### Improvement of cultural conditions

To enhance pectinase production, various physical parameters were determined including cultivation time (14 days with 2 days intervals), temperature (20, 30, 37, 40, and 50 ºC), initial pH (2.0, 4.0, 4.5, 5.0, 6.0, and 8.0), agitation speed (0, 50, 100, 150, 200 and 250 rpm), and inoculums sizes (1 × 10^4^, 1 × 10^5^, 1 × 10^6^, 1 × 10^7^, 1 × 10^8^ and 1 × 10^9^ spores/mL). All the experiments were performed in triplicate.

### Improvement of chemical compositions

Various chemical parameters were improved to enhance pectinase production by *Aspergillus niger* LFP-1. The effect of adding carbon sources such as carboxymethylcellulose (CMC), fructose, glucose, lactose, pectin from citrus fruit, starch, and sucrose with a final concentration of 1.0% (w/v) to the culture medium was determined. The carbon source with maximal pectinase activity from the previous experiment was then investigated at different concentrations [0.5% (w/v), 1.0% (w/v), 1.5% (w/v), 2.0% (w/v), 2.5% (w/v), 3.0% (w/v), and 3.5% (w/v)]. Different inorganic (ammonium chloride, ammonium nitrate, ammonium sulfate, sodium nitrate) and organic (peptone, urea, yeast extract) nitrogen sources were separately supplemented to the culture medium with a final concentration of 0.1% (w/v) also determined. Nitrogen source that showed maximal pectinase production was tested at different concentrations [0.05% (w/v), 0.1% (w/v), 0.2% (w/v), 0.3% (w/v), 0.4% (w/v), 0.5% (w/v), and 0.6% (w/v)]. All the experiments were performed in triplicate.

### Statistical analysis

One-way analysis of variance (ANOVA) and Duncan multiple range tests (DMRT) with PASW (SPSS) Statistics version 12.0 were performed to analyze the significant difference in the mean of experimental data. A 0.05 confidence level or α = 0.05 was used to test all the experimental data.

## Results

### Screening of pectinase production

**Fig. 1:**
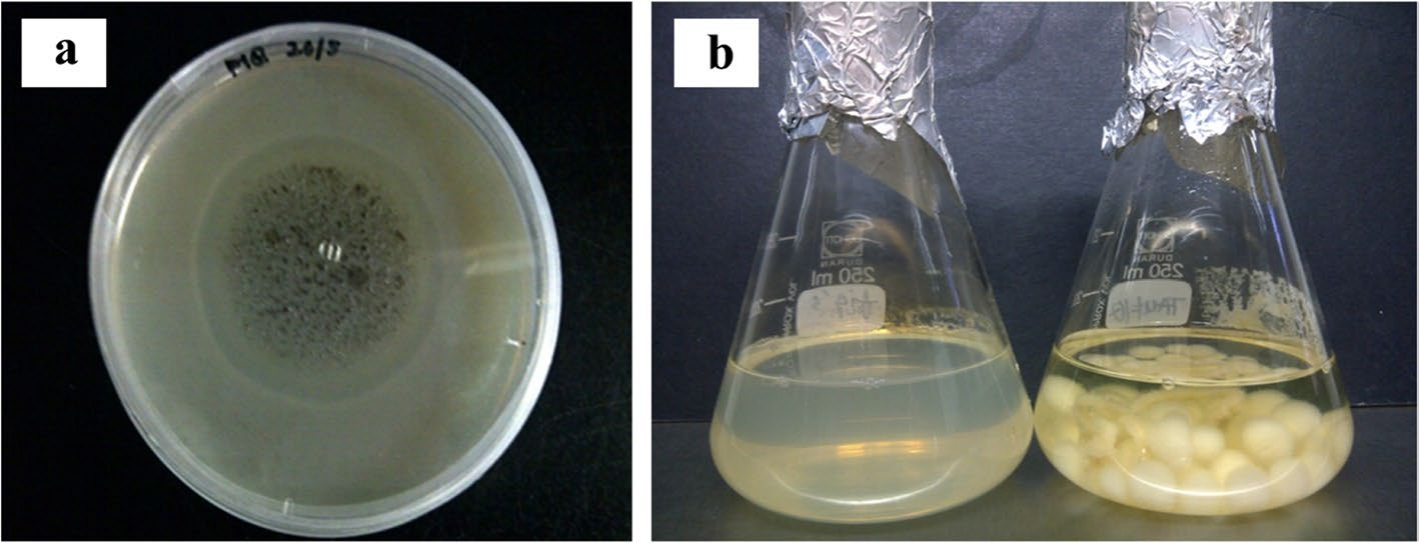
Screening of pectinase activity by *Aspergillus niger* LFP-1. **a** primary screening on pectin agar medium – halo zone indicates the substrate, pectin has been utilized by fungal strain, **b** secondary screening in pectin broth medium – round-shaped fungal cells

Pectinase production by *Aspergillus niger* LFP-1 was screened on pectin agar and pectin broth media and the results are illustrated in Table 1. A clear zone with a diameter of 29.2 ± 0.6 mm was observed on the pectin agar medium. A clear hydrolysis zone that appeared surrounding the active fungal colonies indicates the utilization of pectin by secreting pectinase to produce galacturonic acid as one of its by-products. In addition, a 0.42 ± 0.4 U/mL of pectinase activity was measured through secondary screening on pectin broth media. Besides, Fig. 1 demonstrates the screening of pectinase activity by *A. niger* LFP-1 through primary screening on the pectin agar medium and secondary screening on the pectin broth medium.

**Table 1:** Primary and secondary screening of pectinase activity by *Aspergillus niger* LFP-1

Fungal isolate	Pectinase production	
	**Primary Screening** **(Diameter of clear zone, mm)**	**Secondary Screening** **(U/mL)**
*Aspergillus niger* LFP-1	29.2 ± 0.6	0.42 ± 0.4

### Time course profile of pectinase activity before the enhancement of physicochemical parameters

**Fig. 2:**
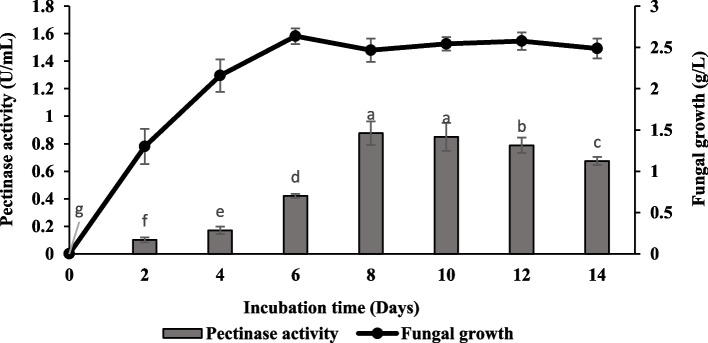
Time course profile of pectinase production in shake flask system by *Aspergillus niger* LFP-1 before improvement of physiochemical parameters [the different letter for each experiment indicated that the results are significantly different (*p* < 0.05)]

Figure 2 demonstrates the growth profile of *Aspergillus niger* LFP-1 and pectinase production in the shake flask system before improved physicochemical parameters. The results showed the pectinase production increased with the cultivation period and achieved its maximal enzyme production on the 8^th^ day of the cultivation period with 0.88 ± 0.09 U/mL and 2.47 ± 0.14 g/L of fungal growth. The pectinase production was consistent until the 10^th^ day of incubation time and then gradually decreased.

### Effect of incubation temperature

**Fig. 3:**
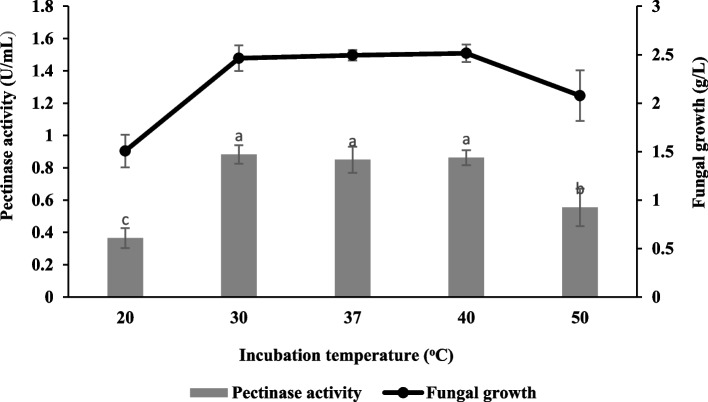
Effect of incubation temperature on pectinase production and fungal growth of *Aspergillus niger* LFP-1 [the different letter for each experiment indicated that the results are significantly different (*p* < 0.05)]

Figure 3 demonstrates that the highest pectinase activity was observed at room temperature (30 ± 2 °C) with 0.88 ± 0.06 U/mL and 2.46 ± 0.13 g/L of fungal growth. The finding also revealed that the pectinase activity at temperatures 37 °C and 40 °C was not significantly different from room temperature with values of 0.85 ± 0.08 U/mL and 0.89 ± 0.05 U/mL, respectively. Besides, temperatures higher (50 °C) or lower (20 °C) than the optimal level reduced pectinase activity and fungal growth.

### Effect of pH

**Fig. 4:**
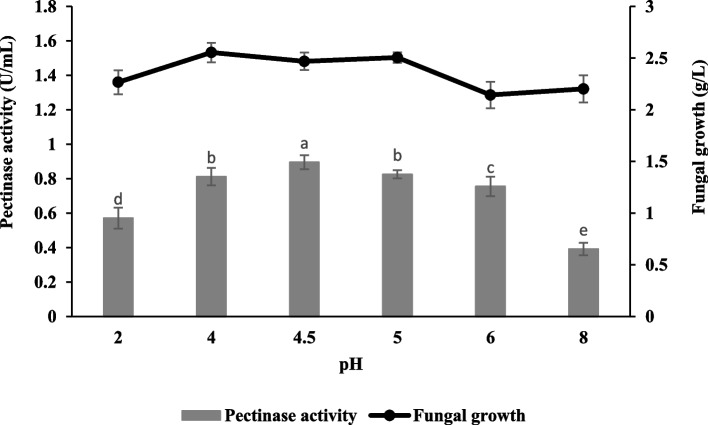
Effect of pH on pectinase production and fungal growth of *Aspergillus niger* LFP-1 [the different letter for each experiment indicated that the results are significantly different (*p* < 0.05)]

The optimal pH for the highest pectinase production by *Aspergillus niger* LFP-1 under submerged fermentation was observed at pH 4.5 with a value of 0.90 ± 0.04 U/mL (Fig. 4). However, the highest fungal growth was achieved at pH 4.0 which was 2.56 ± 0.09 g/L.

### Effect of agitation speed

**Fig. 5:**
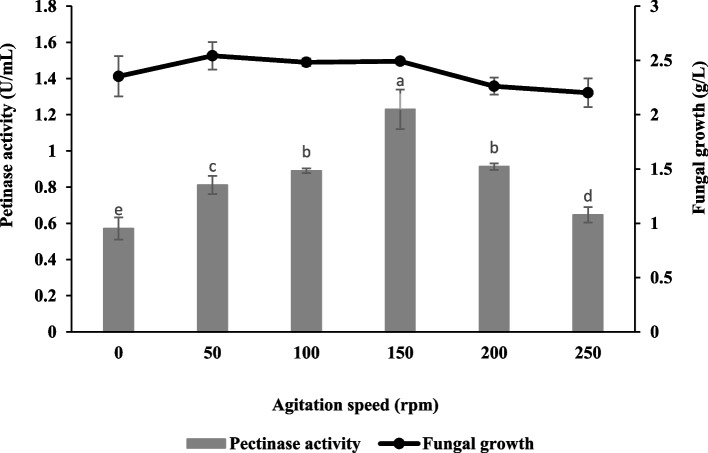
Effect of agitation speed on pectinase production and fungal growth of *Aspergillus niger* LFP-1 [the different letter for each experiment indicated that the results are significantly different (*p* < 0.05)]

Figure 5 exhibits the effect of agitation speed on pectinase production by *Aspergillus niger* LFP-1 and the finding revealed the pectinase activity increased as the agitation speed increased. Maximum pectinase activity was achieved at an agitation speed of 150 rpm with a value of 1.23 ± 0.11 U/mL and the fungal growth was 2.49 ± 0.02 g/L.

### Effect of inoculum size

**Fig. 6:**
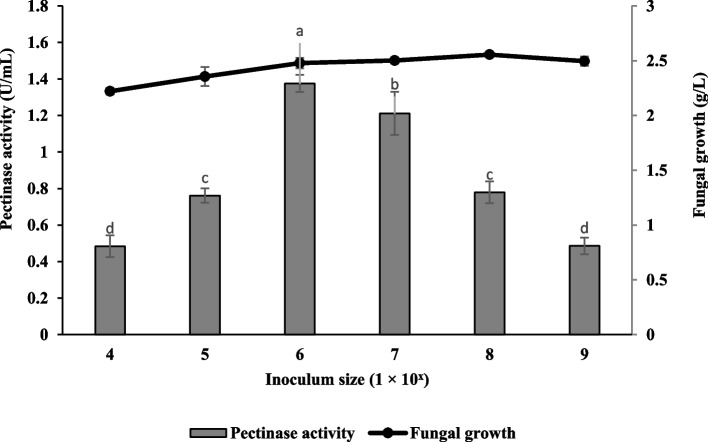
Effect of inoculum size on pectinase production and fungal growth of *Aspergillus niger* LFP-1 [the different letter for each experiment indicated that the results are significantly different (*p* < 0.05)]. Key: *x* indicates 1 × 10^4^, 1 × 10^5^, 1 × 10^6^, 1 × 10^7^, 1 × 10^8^ and 1 × 10^9^

The effect of inoculum size on pectinase production and fungal growth of *Aspergillus niger* LFP-1 was determined. The results are illustrated in Fig. 6. The present study revealed that the highest pectinase production with a value of 1.38 ± 0.05 U/mL (fungal growth, 2.48 ± 0.05 g/L) was achieved at the inoculum size of 1 × 10^6^ spores/mL.

### Effect of carbon sources and their concentrations

**Fig. 7:**
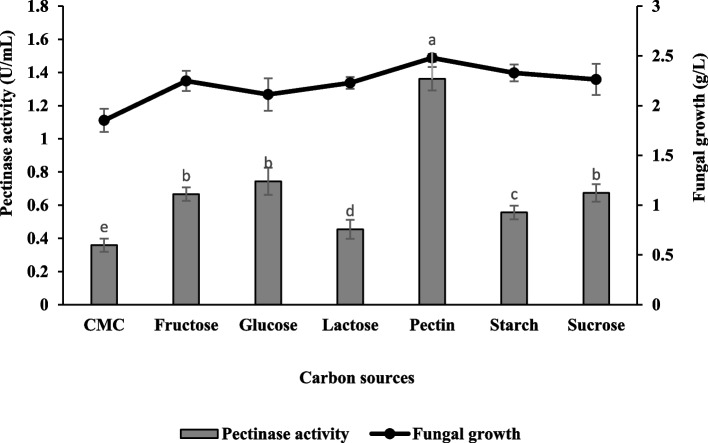
Effect of carbon sources on pectinase production and fungal growth of *Aspergillus niger* LFP-1 [the different letter for each experiment indicated that the results are significantly different (*p* < 0.05)]. Key: CMC – carboxymethyl cellulose

**Fig. 8:**
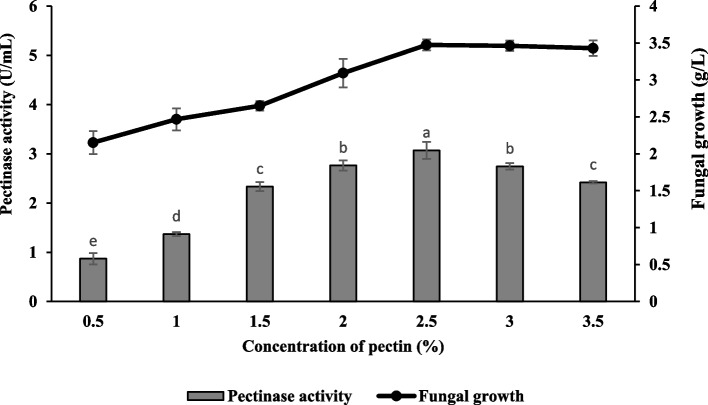
Effect of pectin concentration on pectinase production and fungal growth of *Aspergillus niger* LFP-1 [the different letter for each experiment indicated that the results are significantly different (*p* < 0.05)]

The present study showed citrus pectin was the best carbon source to enhance pectinase production compared to other sole carbon sources studied. The details are presented in Fig. 7. Optimal enzyme production was obtained in the culture medium supplemented with citrus pectin with 1.36 ± 0.07 U/mL and 2.48 ± 0.04 g/L of pectinase activity and fungal growth, respectively. Besides, it is noteworthy that carbon sources such as lactose and CMC significantly suppressed pectinase production with a value of 0.36 ± 0.04 U/mL and 0.45 ± 0.06 U/mL, respectively. The pectinase production was further enhanced by increasing pectin concentration. It was found that citrus pectin at a concentration of 2.5% (w/v) yielded the highest enzyme activity with a value of 3.07 ± 0.17 U/mL (Fig. 8).

### Effect of nitrogen sources and their concentrations

**Fig. 9:**
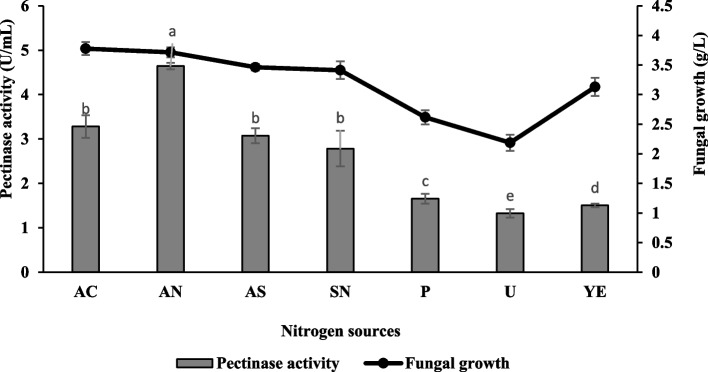
Effect of nitrogen sources on pectinase production and fungal growth of *Aspergillus niger* LFP-1 [the different letter for each experiment indicated that the results are significantly different (*p* < 0.05)]. Key: AC – ammonium chloride, AN – ammonium nitrate, AS – ammonium sulfate, SN – sodium nitrate, P – peptone, U – urea, YE – yeast extract

**Fig. 10:**
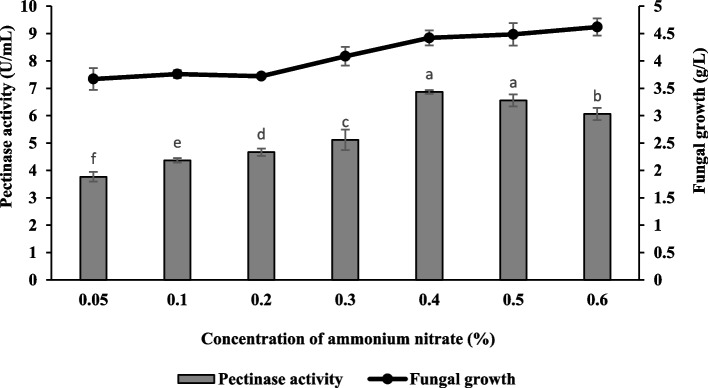
Effect of ammonium nitrate concentration on pectinase production and fungal growth of *Aspergillus niger* LFP-1 [the different letter for each experiment indicated that the results are significantly different (*p* < 0.05)]

In the present study, both organic and inorganic nitrogen sources were studied, and the results were illustrated in Fig. 9. The finding revealed that ammonium nitrate was the best nitrogen source in enhancing pectinase production with the highest enzyme activity being 4.64 ± 0.07 U/mL and 3.72 ± 0.08 g/L of fungal growth. The result also demonstrates that organic nitrogen sources such as peptone, urea, and yeast extract suppressed the pectinase production. The present study concluded that the inorganic nitrogen sources were the best compared to organic nitrogen. The pectinase production was further enhanced by increasing the concentration of ammonium nitrate. It was found that ammonium nitrate at a concentration of 0.4% (w/v) yielded the highest enzyme activity with a value of 6.86 ± 0.07 U/mL (Fig. 10).

### Time course profile of pectinase activity after enhancement of physicochemical parameters

**Fig. 11:**
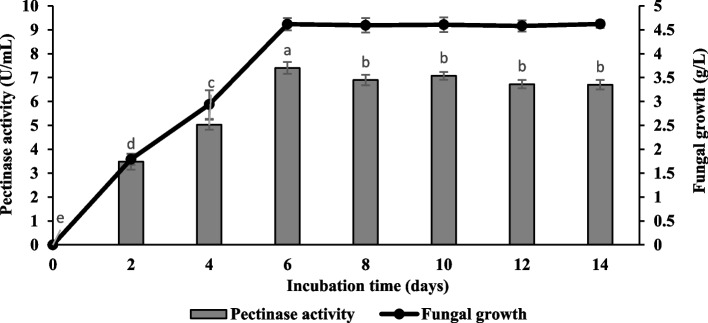
Time course profile of pectinase production in shake flask system by *Aspergillus niger* LFP-1 after improvement of physicochemical parameters [the different letter for each experiment indicated that the results are significantly different (*p* < 0.05)]

A time-course profile was performed for 14 days after the improvement of physicochemical parameters with initial pH was 4.5, incubation temperature of 30 °C, agitation speed of 150 rpm, inoculum size of 1 × 10^6^ spores/mL, 2.5% (w/v) of citrus pectin, and 0.4% (w/v) of ammonium nitrate. Figure 11 shows that pectinase production increased gradually and achieved its optimum production (7.41 ± 0.24 U/mL) after 6 days of cultivation. The pectinase production started to reduce thereafter, and this observation may be due to a shortage of carbon sources and the inhibitory effect of excessive by-products such as polygalacturonic acid. In terms of growth, the fungal biomass increased as the incubation period increased until its maximum growth (4.62 ± 0.08 g/L) after 6 days of cultivation remained almost constant thereafter. Table 2 summarizes the pectinase production and fungal growth by *Aspergillus niger* LFP-1 after improving the physicochemical parameters in a shake flask system. Six parameters were changed including incubation time, inoculum size, agitation speed, the concentration of pectin, nitrogen source, and concentration of nitrogen source. The finding revealed that the pectinase production and fungal growth were significantly increased by 88.12% and 46.54%, respectively after the improvement of physicochemical parameters. It is noteworthy that the improved physical parameters and cultural conditions not only increased the pectinase production but shortened cultivation time from 8 to 6 days.

**Table 2:** The summary of pectinase production and fungal growth by *Aspergillus niger* LFP-1 after improvement of physicochemical parameters in a shake flask system

Parameters	Profiling before improvement	Profiling after improvement
Incubation time (days)	8	6
Incubation temperature (^0^C)	30	30
pH	4.5	4.5
Agitation speed (rpm)	100	150
Inoculum size (spores/mL)	1 × 10^7^	1 × 10^6^
Carbon sources	pectin	pectin
Concentration of pectin (%)	1.0	2.5
Nitrogen sources	ammonium sulfate	ammonium nitrate
Concentration of NH_4_NO_3_	0.1	0.4
Pectinase activity (U/mL)	0.88 ± 0.09	7.41 ± 0.24
Increment (%)	-	88.12
Fungal growth (g/L)	2.47 ± 0.14	4.62 ± 0.13
Increment (%)	-	46.54

## Discussion

The fungal strain, *Aspergillus niger* LFP-1, which was previously collected from the IBRL culture bank was screened for pectinase production using citrus pectin as a substrate by submerged fermentation. The present study used two types of screening methods: primary and secondary screenings. The pectin agar medium was used for primary screening. A clear hydrolysis zone surrounding the active fungal colonies indicates the utilization of pectin by secreting pectinase to produce galacturonic acid as one of its by-products. The findings revealed that the filamentous fungus, *A. niger* LFP-1 can secrete favorable pectinase production. Many previous studies reported the ability of filamentous fungi including *A. niger* to secrete pectinase. For instance, Sethi [10] reported the ability of *A. terreus* NCFT 4269.10 to produce pectinase by utilizing banana peels as a substrate. Meanwhile, Kumar [11] revealed the significant pectinase activity produced by *A. foetidus* through solid-state and submerged fermentation.

Submerged fermentation is influenced by various cultural conditions and growth factors including pH, types of substrates and their concentrations, temperature, inoculum size, agitation speed, and compositions of growth medium. These physicochemical factors have enormous impacts on the production of commercialized enzymes. Other than that, several procedures have been outlined especially for enzyme production produced by microbial species. In the present study, one step at the moment (one variable at a time) was employed for pectinase production by *A. niger* LFP-1 and several cultural conditions were manipulated to increase enzyme yield.

The time-course profile of the growth of *A. niger* LFP-1 cultured in submerged fermentation was monitored for 14 days. The pectinase activity and fungal growth were then determined at an interval of 48 h. Determining the optimal cultivation days with maximal pectinase production is crucial before incorporating physicochemical parameters in this study. The results showed that pectinase production increased with the cultivation period and achieved its maximal enzyme production on the 8th day of the cultivation period. The pectinase production was consistent until the 10^th^ day of incubation time and gradually decreased thereafter. It was suggested that the decrement of pectinase production beyond the maximal level results from nutrient depletion and excessive accumulation of toxic metabolites in the culture medium. The optimum production of enzymes may vary depending on fungal strains and their growth rate, the nature of the culture medium, the concentration of the nutrients, the type of fermentation system, and enzyme production patterns. For instance, Darah [12] reported the maximal production of pectinase by *Aspergillus niger* grown on pomelo peels as a substrate in a solid-state fermentation system on the 6^th^ day of the incubation period. Meanwhile, Ketipally and Ram [13] claimed that the pectinase production by *Aspergillus oryzae* RR 103 in submerged fermentation achieved its maximal activity on the 8^th^ day of the incubation period.

Incubation temperature is one of the crucial factors affecting the metabolic process of various types of cells including promotion/inhibition of the production of a particular metabolite, enzymatic inhibition, protein denaturation, and cell death [14]. Thus, the effect of temperature was investigated in the present study, and enzyme production was improved by increasing the incubation temperature from 20 °C to 30 °C. The result agreed with the previous study that revealed higher pectinase production by *Aspergillus* spp. Gm was observed at an incubation temperature of 30 °C [15]. It is noteworthy that the pectinase production for several temperatures such as 37 °C and 40 °C was not significantly different compared to room temperature (30 ± 2 °C). This indicates that the fungal isolate, *Aspergillus niger* LFP-1 is a mesophilic fungus that is able to grow in a wide range of temperatures. In fact, the fungal isolate was isolated from rotten orange that was discovered in an environment of 30 °C. However, the pectinase production beyond the optimal temperature is significantly lower. According to Ezugwu [16], the increment of pectinase activity as the incubation temperature increased may be due to the changes in conformation that bring the essential residues to proximity for the catalysis process. In contrast, the higher temperature could induce protein denaturation (thermal denaturation). At lower temperatures, the fungal growth might be slow, and delay results less in enzyme production [17]. Even though incubation temperatures of 30 °C, 37 °C, and 40 °C were not significantly different in terms of pectinase activity, the lowest temperature (30 °C) has been chosen to be used in the next experiment. This is because Malaysia is a tropical country with an ambient temperature of 30 °C and thus, by employing this temperature the fermentation cost can be significantly saved up.

The present study revealed that the optimal pH for the highest pectinase production by *Aspergillus niger* LFP-1 under submerged fermentation was observed at pH 4.5; however, the highest fungal growth was achieved at pH 4.0. The results are in line with Sharma and Rishishwar [18] who reported that pectinase production by *A. niger* achieved its maximal activity in the culture medium with an initial pH of 4.5. The result also revealed that pH 4 and 5 showed significant pectinase activity indicating the fungal isolate favours the acidic environment. This phenomenon may be due to the nature of the fungus since it was isolated from rotten orange, which was in the acidic base. According to Fontana and Silveira [19], the pH of a fungal cultivation medium is affected by several conditions including the absorption of nitrogen substances, release of hydrogen ions (H^+^), formation, and consumption of organic acids. The initial pH of the culture medium is crucial since it promotes and regulates the synthesis of the enzyme by microorganisms and thus, the enzyme should be at optimum pH to maximize the velocity of the catalysed reaction [20]. If the pH of the culture medium is below or beyond the optimal level, the velocity is declined, resulting in lower enzyme activity.

Agitation speed significantly affected the pectinase production by *Aspergillus niger* LFP-1 and the finding revealed the pectinase activity increased as the agitation speed increased with maximum pectinase activity obtained at an agitation speed of 150 rpm. Kusuma and Reddy [21] suggested the optimum agitation speed in submerged fermentation would provide sufficient aeration/dissolve oxygen to the culture medium. An increase in mixing speed could help the enzyme's microbial synthesis by increasing sugars' assimilation. Besides, the pectinase production was significantly decreased beyond optimal agitation speed. This phenomenon occurred might be due to the rupture of fungal hyphae due to the high speed of agitation and thus, lead to lower pectinase production. Similarly, Darah [22] suggested that higher agitation speed than the optimum level results in declining pectinase activity due to shear force whereby the collision among the cells occurred and thus, damaged them. Based on a scanning electron microscope (SEM) observation, Ibrahim [23] revealed that the higher agitation speed (200 rpm) than the optimal level resulted in lesser branching mycelia of fungal culture with no formation of fruiting bodies, and this might be reduced pectinase activity. Other than that, lower agitation speed also resulted in lower pectinase activity. This may be due to inadequate dissolved oxygen in the culture medium that delays microbial growth and thus, affects the biosynthesis of the enzyme.

The effect of inoculum size on pectinase production and fungal growth of *Aspergillus niger* LFP-1 was studied and the findings revealed that the highest pectinase production was achieved at the inoculum size of 1 × 10^6^ spores/mL. The finding agreed with the results obtained by Darah [24]. The result also demonstrates that the pectinase activity declined with an increase in inoculum size. Adeyefa and Ebuehi [25] reported that optimum inoculum size is important for the fermentation process since the accumulation of spores can inhibit the growth and development of the microbial culture. Besides, lesser, and greater inoculum density beyond the optimal level resulted in lower pectinase activity. According to Abdullah [17], higher inoculum size leads to excessive accumulation of spores, and competition for nutrients results in less growth as well as enzyme production. An optimal level of inoculum size ensures the rapid proliferation of biomass and enzyme synthesis; thus, lower inoculum density may be inadequate to initiate growth and affect enzyme production.

An adequate supply of carbon sources and their concentrations significantly enhanced fungal growth and its secondary metabolite production. In the present study, citrus pectin was observed as the best carbon source to enhance pectinase production compared to other various sole carbon sources. According to Martos [26], pectin had a positive effect and linear correlation with pectinase activity whereby a higher polygalacturonase (pectinase) production was achieved when the concentration of pectin was increased. On the other hand, it is noteworthy that carbon sources such as lactose and CMC significantly suppressed pectinase production. The result was in line with Rajmane and Korekar [27] who reported that lactose and CMC significantly inhibited pectinase production by test fungi. This phenomenon was suggested due to the inhibitory effect of lactose and CMC on the synthesis of pectinase. A similar observation was reported by Solis-Pereira [28] that revealed the production of polygalacturonase in submerged fermentation was significantly reduced with the addition of free sugars into the culture medium compared to the supplement of pectin as the sole carbon source. In terms of concentration, citrus pectin at a concentration of 2.5% (w/v) yielded the highest enzyme activity and the finding also revealed that the excessive carbon source beyond the optimal level leads to decrement in pectinase activity. Darah [23] suggested that pectinase production was suppressed beyond the maximal level of carbon source due to excessive accumulation of galacturonic acid after hydrolyzation of pectin. This phenomenon might disturb the pH equilibrium in the culture medium and be toxic to fungal growth and thus, affect pectinase activity. Meanwhile, high carbon concentrations may inhibit enzyme synthesis [13].

Nitrogen source and its concentration play a significant role in enzyme production and fungal growth. The present study revealed that ammonium nitrate was the best nitrogen source for enhancing pectinase production. Similarly, Rohit [29] reported the maximum extracellular pectinase of *Aspergillus niger* K3 obtained from the culture medium incorporated with ammonium nitrate. As a comparison, Doughari and Onyebarachi [30] reported optimum pectinase production was observed in the culture medium supplemented with an inorganic nitrogen source, ammonium sulfate. The result also demonstrates that organic nitrogen sources such as peptone, urea, and yeast extract suppressed the pectinase production. The observation contrasts Ire and Vinking [5] who reported the supplement of urea, peptone, and yeast extract strongly induced pectinase produced by *Aspergillus niger*. The present study also concluded that the inorganic nitrogen sources could enhance higher pectinase production compared to organic nitrogen sources. However, this finding was not in line with Vivek [31] who found that culture medium nourished with organic nitrogen sources produced higher pectinase activity compared to inorganic nitrogen sources. In terms of concentration, the minimal concentration of ammonium nitrate could enhance enzyme production. The higher concentration of ammonium nitrate beyond the optimal level resulted in low pectinase production. Excessive ammonium nitrate might inhibit fungal growth or become an inhibitor, thus affecting enzyme production. According to Veverka [32], the growth of fungi was only inhibited by ammonium nitrate in higher concentrations. Overall, adding a nitrogen source into the culture medium significantly enhanced pectinase production. This may be due to microbial cells' utilization of nitrogenous substances for synthesizing proteins, amino acids, nucleotides, enzymes, and other metabolites [33]. In addition, the culture medium incorporated with nitrogen compounds facilitates better biomass production and subsequently increased metabolite secretion [34].

A time-course profile was performed for 14 days after the improvement of physicochemical parameters to access enzyme activity and fungal growth. The pectinase production increased gradually and achieved its optimum after 6 days of cultivation. The pectinase production started to reduce thereafter. This observation may be due to a shortage of carbon sources and the inhibitory effect of excessive by-products such as polygalacturonase acid. Sudeep [15] also reported a decrease in pectinase production beyond the optimum incubation period due to the exhaustion of essential substances and the accumulation of toxic metabolites in the culture medium. The finding revealed that the pectinase production and fungal growth were significantly increased after the improvement of physicochemical parameters, respectively. It is noteworthy that the improved physical parameters and cultural conditions not only increased the pectinase production but shortened cultivation time from 8 to 6 days. A similar observation was reported by Guo [3] who found that fermentation time for pectinase production by *Bacillus* sp. Y1 was shortened 12 h after incorporating improved conditions into the culture medium. Therefore, the shorter fermentation period makes this fungal strain cost-effective for commercial exploitation. Besides, *Aspergillus niger* could be a potential strain to produce pectinase since it has been reported as a very important microbe used in the field of biotechnology, especially in enzyme production including citric acid, amylases, lipases, cellulases, xylanases, and proteases. In addition to that, this strain was also considered generally recognized as safe (GRAS) by the United States Food and Drug Administration and is excepted by the Federal Food, Drug and Cosmetic Act food additive tolerance requirements [35]. Hence, microbial pectinase has a significant potential to be produced for food and beverage-based industries.

## Conclusion

This study demonstrated that the locally isolated fungal strain *Aspergillus niger* LFP-1 is capable of producing pectinase. The improved physicochemical parameters [cultivation period of 6 days, agitation speed of 150 rpm, inoculum size of 1 × 10^6^ spores/mL, 2.5% (w/v) of citrus pectin, and 0.4% (w/v) of ammonium nitrate] have substantially increased pectinase production to 88.12%. The enhanced cultural conditions increase pectinase production and shorten the fermentation time to achieve maximum enzyme production. Further research is required to increase enzyme production by scaling up the bioreactor process. The use of experimental design methods to optimize environmental and nutritional conditions is also recommended. It was suggested that the pectinase be purified and characterized in order to access a particular possible characteristic of a newly isolated enzyme. Since many enzymes produced by *Aspergillus niger* were generally recognized as safe (GRAS), this could be advantageous for the production of fungal enzymes, such as pectinase, for use in the food- and beverage-based biotechnology industries.

## Data Availability

Available on request.
